# Binge Eating and Addictive-Like Eating Behaviors Seven Years After Sleeve Gastrectomy: Implications for Long-Term Weight Loss Outcomes

**DOI:** 10.1007/s11695-026-08751-w

**Published:** 2026-05-20

**Authors:** Tair Ben-Porat, Tamar Avshalom, Hosam Mahajna, Donia Kaloti, Haya Subhi, Ram Elazary, Mahmoud Abu Gazala

**Affiliations:** 1 School of Public Health, Faculty of Social Welfare and Health Sciences, University of Haifa, Haifa, Israel; 2 Faculty of Medicine, Hebrew University of Jerusalem, Jerusalem, Israel; 3 Department of Surgery, Hadassah Medical Center, Jerusalem, Israel

**Keywords:** Obesity, Sleeve gastrectomy, Metabolic bariatric surgery, Binge eating, Addictive-like eating behaviors, Weight recurrence

## Abstract

**Introduction:**

Binge eating (BE) and addictive-like eating behaviors, as assessed by the Yale Food Addiction Scale (YFAS) may change following metabolic bariatric surgery (MBS), yet their long-term trajectories and relationship with weight recurrence remain unclear. We examined their prevalence, longitudinal course, and associations with weight outcomes seven years following sleeve gastrectomy (SG).

**Methods:**

Women who underwent primary SG (*n* = 62) were evaluated prospectively at baseline and at 3, 6, 12, and 24 months, and 7 years post-surgery. Addictive-like eating behaviors and BE were assessed using the YFAS and Binge Eating Scale (BES), respectively. Longitudinal changes and associations with 7-year weight recurrence from nadir were examined.

**Results:**

Thirty women completed the 7-year follow-up. At baseline, addictive-like eating behaviors and BE were identified in 40.0% and 46.0% of participants, respectively. Prevalence declined at 6 months (addictive-like eating behaviors: 8.7%, BE: 10.6%), followed by a gradual increase by 7 years (addictive-like eating behaviors: 21.4%, BE: 26.9%). Baseline behaviors were not associated with weight recurrence at 7 years. In contrast, BE at 7 years was associated with greater weight recurrence from nadir (*P* = 0.015), whereas addictive-like eating behaviors were not.

**Conclusions:**

Although BE and addictive-like eating behaviors decline substantially during the early postoperative period following SG, both may gradually re-emerge over long-term follow-up. BE present at 7 years, but not addictive-like eating behaviors, was associated with significantly greater weight recurrence from nadir, underscoring the importance of sustained behavioral and psychological monitoring during long-term postoperative care.

**Supplementary Information:**

The online version contains supplementary material available at 10.1007/s11695-026-08751-w.

## Key Points

YFAS-defined addictive-like eating behaviors and BE were present in 40–46% of women before SG.

Addictive-like eating behaviors and BE declined early after SG, followed by a gradual increase over time.

Pre-surgical addictive-like eating behaviors and BE were not linked to greater weight recurrence at 7 years.

BE at 7 years, but not addictive-like eating behaviors, was linked to greater weight recurrence from nadir.

**Supplementary Information:**

The online version contains supplementary material available at 10.1007/s11695-026-08751-w.

## Introduction

Sleeve gastrectomy (SG) is currently the most commonly performed metabolic bariatric surgery (MBS) worldwide, and is highly effective for substantial weight loss and metabolic improvement [1, 2]. However, long-term weight recurrence remains a significant clinical challenge, affecting up to 30% of patients, and potentially leading to the re-emergence of obesity-related comorbidities and reduced quality of life [3, 4,5. Although multiple demographic, surgical, metabolic, and behavioral factors have been proposed as contributors, there is still no clear consensus regarding the most influential predictors [3, 4,5.

Maladaptive eating behaviors, including loss-of-control eating, binge eating (BE), and compulsive overeating, are increasingly recognized as key behavioral factors influencing outcomes following MBS [6, 7. BE is clinically well-characterized, classically defined as the consumption of objectively large amounts of food accompanied by a sense of loss of control over eating [8, 9]. Addictive-like eating behaviors, characterized by craving and consumption of highly palatable foods [2, 6, 9, 10], have been well described in the scientific literature and are often referred to as “food addiction”, although this term is not formally recognized by the Diagnostic and Statistical Manual of Mental Disorders (DSM)−5 [9, 11, 12]. The Yale Food Addiction Scale (YFAS) was developed to operationalize this construct by assessing addictive-like eating behaviors based on criteria adapted from substance use disorders; however, it does not constitute a formal clinical diagnosis and reflects a construct that remains debated and the subject of ongoing research [6, 9, 12, 13]. Ultimately, YFAS-defined addictive-like eating behaviors may partially overlap with BE and other maladaptive eating constructs, while remaining conceptually distinct from established clinical diagnoses [6, 9, 13].

Accumulating evidence suggests that YFAS-defined addictive-like eating behaviors and BE are prevalent prior to MBS and decline during the early postoperative period, yet may persist or re-emerge over time [2, 9, 11, 14, 15]. However, existing data are largely limited to short- to mid-term follow-up, typically within the first 1–2 postoperative years, particularly following SG [2, 9, 14, 15]. More recent studies suggest that maladaptive eating behaviors may recur over longer-term follow-up and be associated with less favorable weight maintenance, yet prospective data beyond the early to mid-postoperative phase remain scarce [2, 7, 9–11, 14, 16–18].

We previously reported that addictive-like eating behaviors and BE prevalence decreased significantly during the first postoperative year following SG, and that presurgical BE was associated with less favorable lifestyle patterns and weight outcomes at 2 years post-SG [2, 15]. Importantly, weight recurrence often emerges beyond the early postoperative period, suggesting that short- or mid-term behavioral improvements may not adequately predict long-term weight trajectories [2, 5, 15].

Given the limited evidence on the long-term course of BE and addictive-like eating behaviors, and their potential influence on weight trajectories over extended follow-up periods, the present study aimed to examine the prevalence and longitudinal course of these behaviors, and assess their associations with long-term weight outcomes, including excess weight loss and weight recurrence from nadir, up to seven years following SG.

## Materials and Methods

### Participants

This prospective cohort study represents a long-term extension of a pre-registered randomized controlled trial (RCT; ClinicalTrials.gov Identifier: NCT02483026) that examined the effects of a two-month preoperative vitamin supplementation protocol versus standard pre-surgical care, with 12 months of follow-up, among 62 women undergoing SG [19]. Eating behaviors, lifestyle patterns, and weight outcomes of the combined treatment groups were previously reported for participants who completed 12 months of follow-up, as no significant differences were observed between intervention arms for the primary RCT outcomes [15]. For the present long-term evaluation, all women originally enrolled at baseline were re-invited to participate in a 7-year postoperative follow-up. Of these, 30 women (48.4% of the initial sample) consented and completed the end-point assessment. Thus, the current study extends follow-up by an additional six years beyond the parent trial and reports the long-term course of maladaptive eating behaviors, specifically BE and YFAS-defined addictive-like eating behaviors, and their associations with weight loss outcomes seven years following SG in this prospective cohort.

***Inclusion criteria*** included women aged 18–65 years with BMI ≥ 40 kg/m², or BMI ≥ 35 kg/m² with obesity-related comorbidities. The main ***exclusion criteria*** were untreated mental illness or unstable mental state, pregnancy, lactation, chronic conditions affecting bone metabolism, and a history of MBS [19]. All procedures performed in this study were approved by the institutional review board of Hadassah-Hebrew University Medical Center, and all participants provided written informed consent. Participants were originally recruited between January 2018 and March 2019 and were evaluated at baseline (2 months preoperatively), and at 3 months (3 M), 6 M, 12 M, 24 M, and 7 years (7Y) post-SG. For the current analyses, all women with a measured baseline weight and at least one postoperative weight measurement during the 7-year follow-up period were included.

### Anthropometrics

Weight was measured on a digital medical scale; height was measured by a stadiometer. BMI was calculated as weight (kg) divided by height squared (m²). Percentage of excess weight loss (% EWL) and total body weight loss (%TBWL) were calculated using standard formulas [2, 5]. Weight recurrence was defined as the absolute increase in body weight (kg) from the lowest postoperative weight (nadir) to the 7-year follow-up [5, 20].

### Dietary Intake, Food Tolerance and Physical Evaluation

Participants filled a 3-day food diary to assess macronutrient and micronutrient consumption from food, computed with the Israeli nutritional software “Zameret” [11]. In addition, patients filled a self-reported questionnaire package to assess food tolerance and physical activity level [21].

### BE and Addictive-Like Eating Behaviors Evaluation

The BES is a 16-item self-report instrument commonly used to assess the severity of binge eating symptoms [22]. Among individuals undergoing MBS, the BES has demonstrated high internal consistency (Cronbach’s α = 0.90) [22], as well as strong sensitivity and acceptable specificity for identifying individuals with and without binge eating [22]. Consistent with prior studies, a score > 17 was used to classify participants as likely “binge eaters” [2, 15, 23]. Addictive-like eating behaviors were evaluated using the YFAS, a 25-item self-report questionnaire designed to assess addictive-like eating patterns based on criteria adapted from substance use disorders [6]. The YFAS provides both a dimensional symptom count (number of criteria endorsed) and a dichotomous classification [6]. It provides a proxy measure of addictive-like eating behaviors rather than conferring a formal clinical diagnosis [6, 9, 12, 13].

### Statistical Methods

All statistical analyses were conducted using R software, and data visualization was performed using the *ggplot2* package. Continuous variables are presented as mean ± standard deviation (SD) or median and interquartile range (IQR), as appropriate, and categorical variables as frequencies and percentages. Longitudinal changes in weight outcomes and eating behavior measures across follow-up were examined using repeated-measures linear mixed-effects models. Time, baseline BE and YFAS-defined addictive-like eating behaviors status (yes/no), were specified as fixed effects, with participant included as a random intercept to account for within-subject correlations over time. Changes in the prevalence of BE and addictive-like eating behaviors across repeated time points were assessed using Cochran’s Q test. Comparisons of weight recurrence from nadir between groups at 7-years were performed using the Mann–Whitney U test. All statistical tests were two-sided, and a *P*-value < 0.05 was considered statistically significant.

## Results

### Characteristics of the Participants

Of the 62 women enrolled in the original randomized controlled trial (mean age 32.0 ± 10.6 years) and constituting the baseline cohort of the present study, 54 (87.1%), 36 (58.1%), and 30 (48.4%) completed the 12-month (12 M), 24-month (24 M), and 7-year (7Y, median of 7 years post-SG, IQR = 6.8,7.3) follow-up visits, respectively. YFAS-defined addictive-like eating behaviors data were available for 58 participants at baseline, 51 at 3 months (3 M), 46 at 6 months (6 M), 41 at 12 M, 29 at 24 M, and 28 at 7Y post-surgery. Corresponding binge eating (BES) data were available for 59 participants at baseline, 52 at 3 M, 47 at 6 M, 45 at 12 M, 32 at 24 M, and 26 at 7Y. Baseline characteristics according to preoperative status of BE and addictive-like eating behaviors are presented in Table 1. To assess potential attrition bias, baseline characteristics were compared between participants who completed the 7-year follow-up and those who did not (Table S1). No significant differences were observed between groups across demographic, clinical, or behavioral variables, including binge eating and YFAS-defined addictive-like eating behaviors.

**Table 1:** Baseline characteristics of the study participants according to YFAS-defined addictive-like eating behaviors and binge eating (BE) status prior to sleeve gastrectomy

Parameter†	All participants *n* = 62	Non- Addictive-like eating behaviors *n* = 35 (60%)	Addictive-like eating behaviors *n* = 23 (40%)	*P* - value	Non-BE *n* = 32 (54%)	BE *n* = 27 (46%)	*P* - value
Age (years)	32.0 ± 10.6	31.9±10.5	30.7±10.6	0.6821	31.2±10.7	31.9±10.2	0.7803
Ethnicity [N, (%) Jewish)]	12 (19.3%)	10 (28.6%)	1 (4.4%)	0.0213	9 (28.1%)	2 (7.4%)	0.0418
Marital status [N, (%) married)]	22 (35.5%)	14 (40.0%)	8 (34.8%)	0.6887	14 (43.7%)	8 (29.6%)	0.2638
Education [N, (%) with academic degree]	13 (21.3%)	7 (20.0%)	5 (21.7%)	0.8729	4 (12.5%)	8 (29.6)	0.1034
Monthly income relative to national average (10,634 NIS) [N, (%)]	22 (40.0%)	14 (45.2%)	7 (33.3%)	0.3937	12 (42.9%)	9 (36.0%)	0.6104
Weight (kg)	115.3 ± 14.1	114.9±11.2	118.8±17.1	0.2974	114.1±11.8	118.4±16.1	0.2369
BMI (kg/m^2^)	44.1 ± 4.8	44.6±4.3	44.8±5.5	0.8723	44.4±4.7	44.7±5.2	0.8587
Type 2 diabetes [N, (%)]	7 (11.3%)	3 (8.6%)	2 (8.7%)	0.9868	3 (9.4%)	3 (11.1%)	0.8260
Hypertension [N, (%)]	6 (9.7%)	1 (2.9%)	4 (17.4%)	0.0537	1 (3.1%)	4 (14.8%)	0.1082
Impaired fasting glucose [N, (%)]	30 (49.2%)	17 (48.6%)	10 (43.5%)	0.7037	15 (46.9%)	13 (48.2%)	0.9223
Hypercholesterolemia [N, (%)]	19 (32.2%)	8 (24.2%)	9 (39.1%)	0.2332	10 (33.3%)	8 (29.6%)	0.7639
Hypertriglyceridemia [N, (%)]	20 (33.3%)	10 (29.4%)	10 (43.5%)	0.2750	11 (35.5%)	9 (33.3%)	0.8635
Current smoker [N, (%)]	6 (9.7%)	1 (2.9%)	5 (21.7%)	0.0209	1 (3.1%)	5 (18.5%)	0.0513
BES total score	17.5 ± 8.7	13.2±7.3	24.0±6.7	< 0.0001	11.0±3.8	25.2±6.2	< 0.0001
YFAS-defined Addictive-like eating behaviors total score	3.4 ± 2.0	2.2±1.1	5.3±1.4	< 0.0001	2.2±1.2	4.8±1.8	< 0.0001

†Values are expressed as the mean ± SD or median (IQR) according to the variable. Binary variables are presented as number of patients and percentages. Abbreviations: Binge eating (BE), Binge eating scale (BES), Body mass index (BMI), Yale Food Addiction Scale (YFAS)

### Prevalence of Addictive-Like Eating Behaviors and BE

**Fig. 1:**
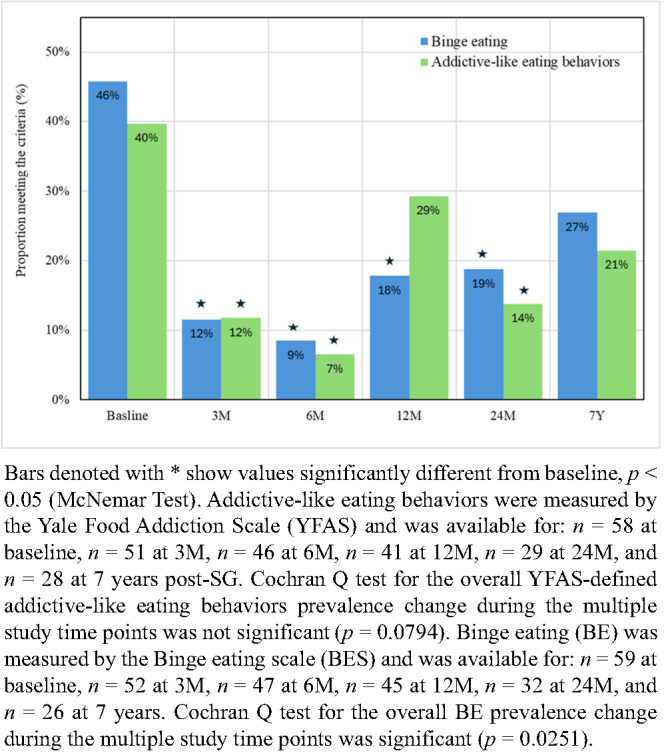
Prevalence of YFAS-defined addictive-like eating behaviors and binge eating before sleeve gastrectomy, and at 3, 6, 12, 24 months (3 M, 6 M, 12 M, 24 M) and 7 years (7Y) postoperative

At baseline, YFAS-defined addictive-like eating behaviors were identified in 23 participants (40.0%) and BE in 27 participants (46.0%). Longitudinal analyses demonstrated that the prevalence of both addictive-like eating behaviors and BE declined markedly during the first postoperative months, followed by a gradual increase over time, with prevalence at 7 years remaining numerically lower than baseline levels (Fig. 1). Overall changes in BE prevalence across follow-up were statistically significant (*P* = 0.025), whereas changes in addictive-like eating behaviors prevalence over time did not reach statistical significance (*P* = 0.079). At 7 years, the prevalence of addictive-like eating behaviors and BE was 21.4% and 26.9%, respectively.

### Weight Loss and Lifestyle Patterns According to baseline status of BE and Addictive-Like Eating Behaviors

Tables 2 and 3 present anthropometric, fitness, and dietary parameters at baseline and across follow-up, according to the presence of BE and YFAS-defined addictive-like eating behaviors prior to SG, respectively. Overall weight trajectories did not significantly differ between women with and without BE at baseline, including weight loss (P-time × group = 0.628), %EWL (P-time × group = 0.357), and %TBWL (P-time × group = 0.295) (Table 2). Similarly, when comparing women with and without addictive-like eating behaviors at baseline, no significant differences in weight trajectories between groups were noted for weight loss (P-time × group = 0.628), %EWL (P-time × group = 0.711), and %TBWL (P-time × group = 0.649) (Table 3). The mean food tolerance score changed significantly over time in both baseline BE and addictive-like eating behaviors groups (P-time < 0.001), with significant time × group interactions indicating greater reductions among participants with presurgical BE and addictive-like eating behavior (P-time × group < 0.050 for both comparisons). Similarly, daily fat intake changed significantly over time, with trajectories differing between participants with and without presurgical BE (P-time × group = 0.035), and between those with and without addictive-like eating behaviors (P-time × group < 0.001), demonstrating an overall higher fat intake over multiple follow-up time points among those with presurgical BE and addictive-like eating behaviors.

**Table 2:** Anthropometrics, dietary behaviors, and lifestyle patterns during the study period according to meeting criteria for binge eating (BE) prior to sleeve gastrectomy

Outcome variable†	Group	Baseline	M3	M6	M12	M24	7-years	*P* time	*P* group	*P* time* group
Non-BE (*n* = 32) compared to BE (*n* = 27) at baseline										
BMI (kg/m^2^)	Non-BE	44.4 ± 4.7	36.0 ± 4.3	32.5 ± 5.0	30.9 ± 5.3	30.8 ± 5.7	34.9 ± 6.8	< 0.0001	0.6227	0.3883
	BE	44.7 ± 5.2	35.5 ± 3.9	31.0 ± 2.7	31.2 ± 5.3	31.7 ± 4.7	34.4 ± 3.7			
Weight loss (kg)	Non-BE	-	−22.7 ± 4.3	−32.0 ± 5.8	−36.1 ± 7.9	−35.8 ± 11.1	−25.5 ± 15.7	< 0.0001	0.9107	0.3427
	BE	-	−24.8 ± 7.0	−32.1 ± 7.6	−36.1 ± 12.8	−31.3 ± 12.3	−24.4 ± 13.5			
EWL (%)	Non-BE	-	−46.3 ± 11.4	−65.6 ± 19.5	−73.8 ± 21.5	−73.4 ± 23.6	−50.8 ± 31.2	< 0.0001	0.4445	0.3566
	BE	-	−47.4 ± 9.1	−67.5 ± 12.1	−70.0 ± 18.9	−64.1 ± 23.0	−47.5 ± 22.3			
TBWL (%)	Non-BE	-	−19.9 ± 3.8	−28.0 ± 5.7	−31.6 ± 7.1	−31.4 ± 9.0	−22.1 ± 13.4	< 0.0001	0.4447	0.2945
	BE	-	−20.6 ± 4.2	−28.2 ± 5.5	−30.2 ± 8.6	−27.1 ± 9.8	−20.2 ± 10.0			
WC (cm)	Non-BE	121.4 ± 11	105.6 ± 9.4	98.6 ± 11.5	93.8 ± 11.7	94.2 ± 13.3	103.6 ± 18.4	< 0.0001	0.5129	0.3942
	BE	124 ± 10.4	105.6 ± 9.8	95.8 ± 9.1	97.8 ± 11.8	97.8 ± 10.7	102.3 ± 9.0			
FT score	Non-BE	24.8 ± 1.7	22.1 ± 2.9	22.8 ± 3.0	22.6 ± 3.8	22.4 ± 3.3	22.7 ± 3.4	< 0.0001	0.0878	0.0062
	BE	25.5 ± 1.3	19.8 ± 3.8	21.3 ± 4.0	20.1 ± 4.3	22.8 ± 3.6	22.0 ± 2.9			
Calories (kcal/d)	Non-BE	3029.3 ± 349.2	907.9 ± 179.8	937 ± 161.6	1086.2 ± 238.5	1080.5 ± 216.4	1334.6 ± 880.4	< 0.0001	0.3321	0.3086
	BE	3271.4 ± 751.4	878.3 ± 190.6	932.4 ± 146.7	1112.6 ± 285.6	1050.8 ± 237.4	1493.6 ± 708.6			
Protein (gr/d)	Non-BE	124.2 ± 28.0	50.8 ± 14.9	51.3 ± 13.5	56.2 ± 15.5	56.7 ± 23.1	61.6 ± 26.6	< 0.0001	0.4649	0.6141
	BE	128.3 ± 41.8	48.8 ± 13.2	52.3 ± 18.5	53.0 ± 13.0	54.8 ± 20.5	76.6 ± 33.1			
Carbs (gr/d)	Non-BE	385.8 ± 68.1	97.3 ± 34.0	98.3 ± 22.2	114.1 ± 50	117.9 ± 31.4	150.3 ± 120.9	< 0.0001	0.7652	0.9704
	BE	394.1 ± 107.5	100.1 ± 30.2	106.3 ± 30.3	124.7 ± 43.4	108.3 ± 38.5	150.3 ± 100.7			
Fat (gr/d)	Non-BE	108.7 ± 31.5	35.2 ± 13.5	38.3 ± 12.5	42.7 ± 13.5	40.2 ± 11.4	52.0 ± 37.6	< 0.0001	0.1196	0.0346
	BE	130.7 ± 44.8	33.4 ± 10.9	35.0 ± 10.3	46.8 ± 16.8	42.4 ± 12.5	62.1 ± 31.1			

†Data are presented as estimated mean (SE) according to the mixed model analysis [P time- Time effect = analyze the changes over time in the two groups; P groups- Group effect= analyze between groups differences; P time*group- Group time effect = analyze the interaction between the trend of change over time and the group effect]. Rates with different superscripts (^a, b^) differ significantly from each other in that row for within group differences at *P* ≤ 0.05. Abbreviations: Binge eating (BE), Body mass index (BMI), Carbohydrates (Carbs), Excess weight loss (EWL), Food tolerance (FT), Waist circumference (WC), Total body weight loss (TBWL), Three, 6, 12, 24 months and 7-years postoperative (M3, M6, M12, M24, 7 years)

**Table 3:** Anthropometrics, eating behaviors and lifestyle patterns during the study period according to meeting criteria for YFAS-defined addictive-like eating behaviors prior to sleeve gastrectomy

Outcome variable†	Group	Baseline	M3	M6	M12	M24	7-years	*P* time	*P* group	*P* time* group
Non- Addictive-like eating behaviors (*n* = 35) compared to Addictive-like eating behaviors (*n* = 23) at baseline										
BMI (kg/m^2^)	Non-AE	44.6 ± 4.3	35.9 ± 4.1	32.5 ± 4.8	31.5 ± 5.2	31.6 ± 5.8	34.8 ± 6.5	< 0.0001	0.8934	0.6516
	AE	44.8 ± 5.5	35.5 ± 4.1	30.8 ± 2.8	30.5 ± 5.4	30.7 ± 4.3	34.5 ± 3.2			
Weight loss (kg)	Non-AE	-	−22.5 ± 4.2	−31.4 ± 6.1	−34.4 ± 8.6	−33.6 ± 12.1	−25.3 ± 14.9	< 0.0001	0.3330	0.6284
	AE	-	−25.5 ± 7.3	−33 ± 7.2	−38.5 ± 12.4	−33.8 ± 11.6	−24.4 ± 14.3			
EWL (%)	Non-AE	-	−45.9 ± 11	−64.9 ± 19.2	−70.3 ± 22.1	−68.9 ± 25.4	−50.7 ± 30.1	< 0.0001	0.7734	0.7111
	AE	-	−48.3 ± 9.2	−68.9 ± 11.4	−74.3 ± 17.2	−69.4 ± 21.2	−46.7 ± 21.8			
TBWL (%)	Non-AE	-	−19.6 ± 3.6	−27.6 ± 5.8	−30.1 ± 7.7	−29.4 ± 10.1	−21.9 ± 12.8	< 0.0001	0.6646	0.6492
	AE	-	−21.1 ± 4.3	−28.9 ± 5.2	−32.1 ± 7.9	−29.3 ± 9	−20 ± 10.1			
WC (cm)	Non-AE	121.9 ± 10	105.3 ± 9.4	98.7 ± 11.1	94.9 ± 11.7	96.2 ± 13.1	103.4 ± 16.8	< 0.0001	0.9534	0.4898
	AE	124.7 ± 10.7	106 ± 9.9	95.3 ± 9.4	96.9 ± 12.2	95.4 ± 10.7	102.2 ± 9.1			
FT score	Non-AE	24.9 ± 1.7	21.8 ± 2.8	22.3 ± 3.4	22.5 ± 3.8	22.5 ± 3.2	22.7 ± 3.2	< 0.0001	0.1523	0.0068
	AE	25.5 ± 1.2	19.8 ± 4.2	21.8 ± 3.8	19.8 ± 4.4	22.7 ± 3.9	21.7 ± 3.1			
Calories (kcal/d)	Non-AE	3002.2 ± 340	873.5 ± 157.6	946.5 ± 150.5	1117.4 ± 247.6	1089.6 ± 225.5	1293.2 ± 819.8	< 0.0001	0.1340	0.0195
	AE	3340.4 ± 790.6	926.4 ± 217.6	917 ± 161.1	1071.6 ± 281.2	1029.2 ± 225.1	1611.8 ± 736.2			
Protein (gr/d)	Non-AE	122.6 ± 26.4	49.4 ± 15	53.1 ± 16.2	57.4 ± 14.7	56.5 ± 21.2	61.8 ± 27.6	< 0.0001	0.3209	0.1741
	AE	132.3 ± 45	50.6 ± 12.8	49.7 ± 15.1	50.7 ± 12.9	54.5 ± 23	80.8 ± 32.4			
Carbs (gr/d)	Non-AE	384.8 ± 66.8	94.6 ± 32.8	97.9 ± 22.4	118.5 ± 48.6	117 ± 33.8	145.2 ± 111.4	< 0.0001	0.5586	0.9377
	AE	393.6 ± 114.1	104.6 ± 30.7	107.9 ± 30.5	120.2 ± 45.1	107.2 ± 36.9	158.9 ± 111.5			
Fat (gr/d)	Non-AE	107.8 ± 32.2	33.3 ± 11.4	39.2 ± 11.9	44.3 ± 14.1	41.5 ± 12	49.4 ± 35.2	< 0.0001	0.0459	0.0009
	AE	135.2 ± 44.9	36 ± 13.7	33.3 ± 10.4	45.1 ± 17	40.9 ± 12	69.5 ± 30.2			

†Data are presented as estimated mean (SE) according to the mixed model analysis [P time- Time effect = analyze the changes over time in the two groups; P groups- Group effect= analyze between group differences; P time*group- Group time effect = analyze the interaction between the trend of change over time and the group effect]. Rates with different superscripts (^a, b^) differ significantly from each other in that row for within group differences at *P* ≤ 0.05. Abbreviations: Addictive-like eating behaviors (AE), Body mass index (BMI), Carbohydrates (Carbs), Excess weight loss (EWL), Food tolerance (FT), Waist circumference (WC), Total body weight loss (TBWL), Three, 6, 12, 24 months and 7 years postoperative (M3, M6, M12, M24, 7 years), Yale Food Addiction Scale (YFAS)

### Pre- and Post-Surgical BE and Addictive-Like Eating Behaviors, and Weight Recurrence

**Fig. 2:**
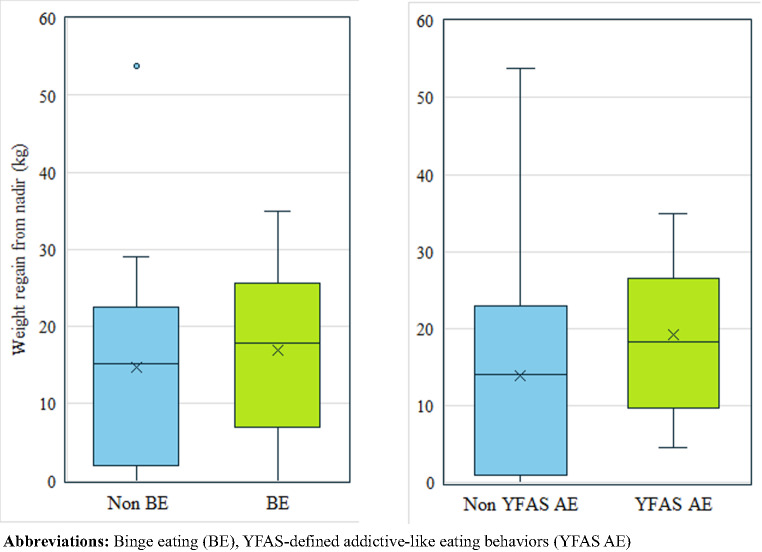
Recurrent weight gain from the nadir at 7 years according to meeting the criteria for binge eating [A] and YFAS-defined addictive-like eating behaviors [B] prior to sleeve gastrectomy

**Fig. 3:**
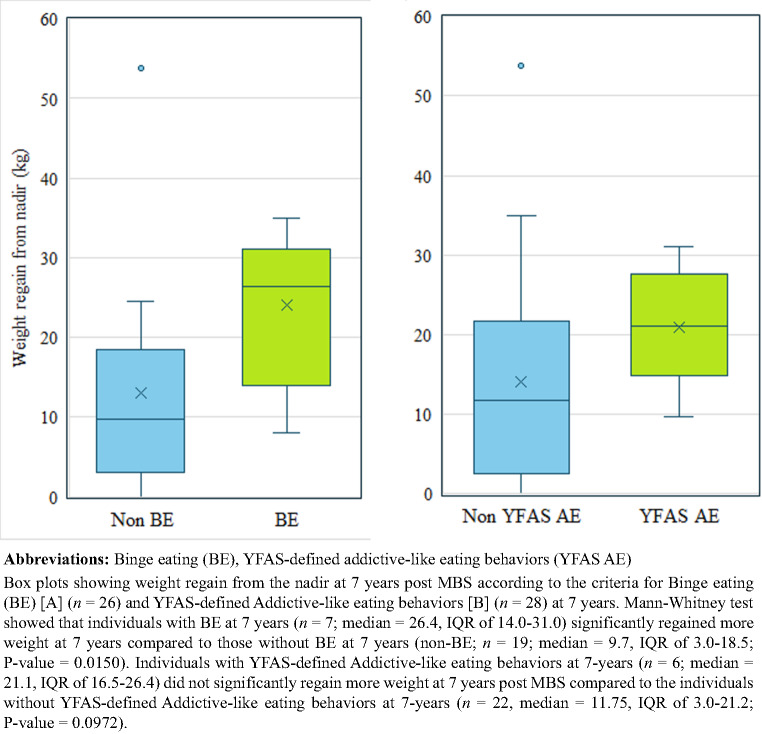
Recurrent weight gain from the nadir at 7 years according to meeting the criteria for binge eating [A] and YFAS-defined addictive-like eating behaviors [B] at 7 years

The mean postoperative nadir weight was 75.3 ± 11.5 kg, reached at a median of 18.0 months (IQR = 12.0, 53.3) following surgery. Women with BE or YFAS-defined addictive-like eating behaviors at baseline did not experience significantly greater weight recurrence from nadir at 7 years compared with those without BE or addictive-like eating behaviors (*P* = 0.323 and *P* = 0.117, respectively) (Fig. 2). In contrast, participants meeting criteria for BE at the 7-year follow-up demonstrated significantly greater weight recurrence from nadir compared with those without BE (*P* = 0.015) (Fig. 3). No statistically significant association was observed for addictive-like eating behaviors at 7 years (*P* = 0.097).

Box plots showing weight regain from the nadir at 7 years post MBS according to meeting the criteria for Binge eating (BE) [A] and YFAS-defined Addictive-like eating behaviors [B] at baseline (*n* = 29). Mann-Whitney test showed that individuals with BE at baseline (*n* = 13; median = 17.9, IQR of 9.5–25.0) did not significantly regain more weight at 7 years compared to those without BE at baseline (non-BE; *n* = 16; median = 15.2, IQR of 3.0–22.1.0.1; P-value = 0.3230). Individuals with YFAS-defined addictive-like eating behaviors at baseline (*n* = 10; median = 18.2, IQR of 9.7–25.0) also did not significantly regain more weight at 7 years post MBS compared to the individuals without YFAS-defined addictive-like eating behaviors at baseline (*n* = 19, median = 14.0, IQR of 1.0–23.0; P-value = 0.1168).

## Discussion

Several key findings emerge from this long-term follow-up across women who underwent SG. First, the prevalence of both BE and addictive-like eating behaviors declined markedly during the early postoperative period, followed by a gradual increase over time, with prevalence at 7 years remaining numerically lower than baseline levels. Second, presurgical BE and addictive-like eating behaviors were not significantly associated with long-term weight recurrence from nadir. In contrast, the presence of BE at 7 years was significantly associated with greater weight gain, highlighting the potential clinical relevance of ongoing maladaptive eating behaviors during long-term postoperative follow-up. Importantly, these findings should be interpreted in light of the different nature of the constructs examined: binge eating is a clinically recognized syndrome with established diagnostic criteria and clear clinical relevance, whereas YFAS-defined addictive-like eating behaviors represent a debated research construct [6, 9, 12, 13].

The marked early postoperative decline in both BE and addictive-like eating behaviors observed in the present study is consistent with previous reports demonstrating substantial reductions in problematic eating behaviors during the first postoperative year following MBS [9–11, 14, 16]. The decreased prevalence of BE postoperatively may be explained by the restrictive nature of SG and the strong physiological effects of surgery during the early postoperative phase, including reduced gastric capacity, altered satiety signaling, and changes in appetite-regulating gut hormones [11, 24]. Over time, however, the restrictive physiological effects of SG may attenuate, progressively reducing the procedural constraints that initially suppress maladaptive eating behaviors, while some re-emergence of these behaviors may occur, potentially reflecting behavioral adaptation to the procedure, attenuation of physiological effects, or challenges in sustaining long-term lifestyle changes and coping strategies [4, 5, 8, 10, 13, 15, 16, 25]. As a result, underlying or re-emerging behavioral patterns, particularly BE, may become more prominent and exert a greater influence on long-term weight trajectories [4, 5, 8, 10, 13, 15, 16, 25]. Similarly, the decrease in addictive-like eating behaviors during the first postoperative year concurs with prior reports showing postoperative prevalence rates ranging from 2% to 21% [14, 16, 26]. Importantly, the present study extends these earlier findings by demonstrating a gradual increase in both BE and addictive-like eating behaviors prevalence across longer-term follow-up. In that regard, our findings align with more recent long-term literature: Smith et al. reported recurrence and persistence of loss-of-control and BE behaviors across 7 years following MBS [7]. Similarly, Reas et al. have recently demonstrated at 10-year follow-up, that BE symptoms may remit, persist, recur, or emerge de novo over time [17]. In addition, Lie et al. showed that addictive-like eating behaviors remain clinically relevant even at 5- and 10-years following MBS [18]. Taken together, these data, alongside the present findings, suggest that early postoperative remission of problematic eating behaviors should not be interpreted as their sustained long-term resolution.

An important finding of the present study is that presurgical BE and addictive-like eating behaviors were not significantly associated with long-term weight recurrence from nadir at 7 years. One possible interpretation is that presurgical behavioral status may have greater relevance to short- and mid-term outcomes, whereas long-term weight maintenance may be more strongly influenced by behavioral patterns that persist, emerge, or recur over time [7, 9, 12]. Indeed, one of the most clinically relevant findings of the present study is that BE present at the 7-year follow-up was associated with significantly greater weight recurrence from nadir. This finding suggests that BE may not only represent a presurgical vulnerability factor but may also reflect ongoing behavioral patterns that may be associated with long-term weight regain following SG. These findings are further supported by recent literature [17, 27. Reas et al. similarly reported that persistent and de novo BE at 10 years was associated with poorer long-term weight maintenance [17]. Likewise, Cali et al. recently demonstrated that BE behaviors following MBS were associated with recurrent weight gain [27]. In contrast to BE, addictive-like eating behaviors post-SG did not show a statistically significant association with long-term weight outcomes in the present cohort, which is consistent with several previous studies, suggesting that the clinical implications of addictive-like eating behaviors following MBS remain less clear [2, 9]. Collectively, these findings suggest that addictive-like eating behaviors may reflect a distinct psychological or behavioral vulnerability phenotype that does not translate into long-term weight trajectories in the same manner as BE [2, 9, 12]. This distinction reinforces the clinical relevance of BE for long-term outcomes, while supporting the interpretation that YFAS-defined addictive-like eating behaviors might represent a related but conceptually distinct and less clinically established construct [6, 9,13. Overall, these results add to growing evidence that maladaptive eating behaviors assessed concurrently, rather than preoperatively, may be more strongly associated with long-term weight outcomes following MBS.

To the best of our knowledge, this is among the few prospective SG cohorts to examine maladaptive eating behaviors over as long as 7 years with multiple postoperative time points. The prospective design, repeated assessments, and use of objectively measured clinic weights rather than self-reported data strengthen the validity of the long-term behavioral and weight outcomes, and enabled characterization of longitudinal trajectories over time. Several limitations should nevertheless be acknowledged. First, the sample size at the 7-year follow-up was modest, which may have limited statistical power, particularly for subgroup and addictive-like eating behaviors-related analyses. In that regard, it is important to note that the number of participants meeting criteria for YFAS-defined addictive-like eating behaviors at 7 years was small, which might have limited the statistical power for this subgroup analysis. Accordingly, the absence of a significant association with weight recurrence should be interpreted with caution, and these findings should be considered preliminary and hypothesis-generating. Second, only women were included, which limits generalizability of the findings to male populations. Thus, although women represent the majority of patients undergoing MBS worldwide, future studies should probably include men to determine whether these findings extend across sexes. In addition, baseline ethnicity differed between groups; however, analyses adjusted for ethnicity did not materially alter the results, suggesting that these differences were unlikely to account for the findings. Additional psychological variables such as depression, anxiety, and broader eating psychopathology were not assessed longitudinally, and future studies should further examine their role in long-term weight recurrence. It should also be noted that the term “food addiction,” as used in prior studies, typically refers to YFAS-derived outcomes and reflects a research construct rather than a formal diagnosis. Finally, although loss to follow-up is an inherent challenge in long-term bariatric cohorts, baseline comparisons between completers and non-completers did not reveal significant differences in key demographic, clinical, or behavioral variables, suggesting a relatively low risk of selection bias. Taken together, this study’s findings support the importance of ongoing behavioral monitoring as part of routine postoperative care. Specifically, repeated screening for maladaptive eating behaviors using validated instruments may be warranted beyond the early postoperative period, as behavioral improvements observed during the first year may not be sustained over time.

**In conclusion**, the present study demonstrates that although BE and addictive-like eating behaviors decline substantially during the early postoperative period following SG, both behaviors may gradually re-emerge over long-term follow-up. Importantly, BE present at 7 years, rather than presurgical behavioral status, was associated with significantly greater weight recurrence from nadir. These findings underscore the importance of sustained behavioral and psychological monitoring throughout long-term postoperative care and suggest that continued multidisciplinary follow-up may be essential for optimizing long-term weight outcomes.

## Supplementary Information

Below is the link to the electronic supplementary material.

Supplementary file 1 (DOCX 56.0 KB)

## Data Availability

The datasets generated and/or analyzed during the current study are not publicly available due to participant privacy and institutional ethical restrictions. Certain study-related materials are available from the corresponding author upon reasonable request and subject to approval by the relevant institutional review board.
