# Supplementary material for: Binge Eating and Addictive-Like Eating Behaviors Seven Years After Sleeve Gastrectomy: Implications for Long-Term Weight Loss Outcomes
Source: Obes Surg. 2026 May 20;36(7):3675–84. doi: 10.1007/s11695-026-08751-w (PMC13323189; doi:10.1007/s11695-026-08751-w)
Supplement: Supplementary file 1 — (DOCX 56.0 KB) [file 11695_2026_8751_MOESM1_ESM.docx]

**Table S1.** Baseline characteristics of participants according to 7-year follow-up status (completers vs. non-completers)

| Variable† | Completers (n=30) | Non-completers (n=32) | P-value |
| --- | --- | --- | --- |
| Age (years) | 34.1 ± 10.5 | 30.1 ± 10.5 | 0.144 |
| BMI (kg/m²) | 44.3 ± 4.4 | 44.5 ± 5.3 | 0.845 |
| Weight (kg) | 113.0 ± 12.0 | 113.2 ± 15.7 | 0.962 |
| BES score | 16.2 ± 7.4 | 18.8 ± 9.8 | 0.252 |
| Binge eating (yes, %) | 13/29 (44.8%) | 14/30 (46.7%) | 0.887 |
| YFAS total score | 3.4 ± 2.1 | 3.5 ± 1.8 | 0.843 |
| YFAS-defined addictive-like eating  behaviors (yes, %) | 10/29 (34.5%) | 13/29 (44.8%) | 0.421 |
| Food tolerance score | 25.1 ± 1.4 | 25.2 ± 1.7 | 0.866 |
| Ethnicity (Arab) | 24 (80.0%) | 26 (81.3%) | 0.901 |
| Education (academic degree) | 8 (26.7%) | 5 (15.6%) | 0.315 |
| Monthly income ≥ national average | 11 (36.6%) | 11 (34.3%) | 0.741 |
| Type 2 diabetes | 5 (16.7%) | 2 (6.3%) | 0.195 |
| Hypertension | 4 (13.3%) | 2 (6.3%) | 0.346 |
| Impaired fasting glucose | 13 (43.3%) | 17 (53.1%) | 0.369 |
| Steatosis | 25 (83.3%) | 27 (84.4%) | 0.911 |
| OSAP | 1 (3.3%) | 2 (6.3%) | 0.593 |
| Psychiatric diagnosis | 1 (3.3%) | 2 (6.3%) | 0.593 |
| Current smoker | 2 (6.7%) | 4 (12.5%) | 0.438 |
| Statin use | 4 (13.3%) | 2 (6.3%) | 0.346 |
| Psychiatric medication use | 1 (3.3%) | 2 (6.3%) | 0.593 |
| Diabetes medications use | 2 (6.7%) | 2 (6.3%) | 0.947 |

†Continuous variables are presented as mean ± standard deviation (SD). Categorical variables are presented as n (%).

**Abbreviations:** BMI, body mass index; BES, Binge Eating Scale; YFAS, Yale Food Addiction Scale; OSAP, obstructive sleep apnea.
